# A "radical" mitochondrial view of autophagy-related pathology

**DOI:** 10.18632/aging.100037

**Published:** 2009-04-08

**Authors:** Nuno Raimundo, Gerald S. Shadel

**Affiliations:** ^1^ Department of Pathology, Yale University, School of Medicine, New Haven, CT 06520, USA; ^2^ Department of Genetics, Yale University, School of Medicine, New Haven, CT 06520, USA

**Keywords:** autophagy, mitochondria, reactive oxygen species, pathology, respiration, β-cell, mTOR, Atg7

Autophagy is a critical catabolic process through which cytoplasmic components are degraded, with the primary purpose of removing long-lived and/or damaged proteins and organelles and recycling essential anabolic building-block molecules [1]. Mechanistically, this involves formation of autophagosomes that are fused with lysosomes to facilitate digestion of the internalized components by lysosomal proteases. Proteins that mediate specific steps of the autophagy pathway have been characterized, mutations in which cause severe disease pathology affecting tissues such as muscle, brain, heart, liver and the immune system [2].  In addition, defects in autophagy are associated with aging and age-related pathology in yeast, worm and fly model organisms [3-6].

In mammals, autophagy is essential, as demonstrated by neonatal lethality of specific gene knock-outs of proteins required for the process, underscoring its importance in cell homeostasis, as well as organismal growth and development [1]. Conditional and tissue-specific knock-out of genes involved in autophagy in mice have provided more detailed insight into this process.  For example, such approaches have uncovered that impaired autophagy results in phenotypes and pathology that are commonly observed during the normal aging process [1].  While the precise underlying mechanisms driving autophagy-related pathology remain obscure, the study of Finkel and colleagues  (Wu et al.,  in this issue of *Aging*) provides critical new insight by showing that mitochondrial dysfunction is likely a critical precipitating factor.

In their study, Wu et al. show that impairment of autophagy, via deletion of the Atg7 gene that is essential for the process, profoundly influences mitochondrial function.  In a skeletal muscle-specific Atg7 knock-out, ultra-structural abnormalities in mitochondria are observed, as is decreased basal and maximal respiration due to impairments of complex I and II activity. This result was largely reproduced in mouse embryonic fibroblasts from Atg7-/- embryos, which also display markedly reduced basal respiratory activity and total mitochondrial oxidative capacity. These effects appear to be levied at the level of respiration activity directly, as opposed to through effects on overall mitochondrial biogenesis, because no differences in the amounts of assembled oxidative phosphorylation complexes or amount of mtDNA/cell (a common measure of mitochondrial abundance) were observed.  The lack of an overt physiological phenotype in the skeletal muscle of these mice appears to be due, at least in part, to a compensatory increase in glycolysis.  Importantly, the lower mitochondrial respiratory activity is associated with an increase in steady-state levels of cellular reactive oxygen species (ROS).  It remains to be determined whether this increase is due to increased mitochondrial ROS production, a decrease in cellular antioxidant defenses, or both.  Nonetheless, Wu *et al.* demonstrate that treatment of Atg7-/- MEFs with the antioxidant N-acetylcysteine (NAC) reverts the mitochondrial phenotypes, both in terms of respiration and ROS.  Perhaps as expected, the antioxidant treatment did not restore autophagy *per se*, suggesting that ROS are key downstream mediators of mitochondrial dysfunction in the absence of autophagy. This conclusion is bolstered by the recent study by Tal et al. [7], who also showed that ROS are downstream mediators of impaired autophagy.  However, in this case, lack of autophagy due to Atg5 knock-out results in ROS-dependent enhancement of innate antiviral signaling (i.e. RLR signaling) and interferon production and, consequently, the beneficial effect of providing resistance to a viral infection.  Together, these two studies highlight 1) the complex and tissue-specific role that ROS play in cells, acting as both signaling molecules and mediators of oxidative damage and 2) that perturbation of ROS homeostasis is a key cellular event that follows down-regulation of autophagy.

In their study, Wu et al. also created mice with pancreatic β-cell-specific deletion of Atg7.  While the insulin expression and the overall cellular structure of the pancreatic islets in these mice at 8-weeks of age are largely unperturbed, abnormal mitochondrial morpho-logy and respiration are apparent. This is accompanied by oxidative stress and the previously described disturbance of glucose tolerance and insulin secretion.  Similar to the results obtained in autophagy-minus skeletal muscle, Wu and colleagues found that *in vivo* NAC treatment reverted the oxidative stress-related phenotypes in β-cells lacking autophagy, while not reverting the autophagy defect *per se*.  In addition, NAC treatment also corrected the glucose tolerance and insulin secretion defects, again implicating ROS as a central downstream mediator of the cellular and physiological responses to faulty autophagy.

While the study of Wu *et al.*, and others [7] solidify the role of ROS in mediating the effects of debilitated autophagy, it is likely that other mitochondrial-derived mediators (e.g. calcium, ATP/ADP, other metabolites) are involved. And, as pointed out by Wu et al., these are likely to be tissue-specific and/or different depending on the specific defect in the autophagic machinery that is causative.  Furthermore, it is important to stress that the work of Finkel and colleagues demonstrates that mitochondrial abnormalities occur at a very early stage, when no pathology is yet evident in the tissues, which very likely places mitochondrial perturbations at the core of the disease pathology caused by certain autophagy defects.  It will be of great interest to deter-mine if mitochondrial signals (ROS or otherwise) are instructive in promoting the up-regulation of glycolysis and other metabolic alterations and which signaling pathways are reading out these directly.  In this regard, the role of the nutrient-sensing, target of rapamycin (TOR) pathway in responding to and controlling mitochondrial respiration [8-11] is intriguing, especially since TOR kinase also has its hands in controlling autophagy [12] and is potentially a redox-sensitive enzyme that could directly respond to altered ROS [13].

Finally the study by Wu et al. opens many new avenues of future investigation that could have a positive impact on potential therapeutic routes for autophagy-related diseases and age-related pathology. Certainly, their results suggest that antioxidant treatment may be a viable option to pursue further. This, however, is likely to be a slippery slope given the aforementioned signaling role for ROS.  That is, antioxidants may be valuable for extreme cases of oxidative stress-related pathology, but may, at the same time, perturb other pathways in unpredictable ways that could cause unwanted side effects. In a larger context, the Wu et al. study joins a rapidly growing list that shows mito-chondrial dysfunction lies at the center of not only rare metabolic diseases, but also common ailments that plague our society such as diabetes, heart disease, neurodegenerative disorders, and age-related pathology. Determining the nature of the connections between mitochondrial dysfunction, ROS and pathogenic mechanisms is a burgeoning area of great importance. Precise modulation of the autophagy pathway appears to be a novel route toward understanding these connections and perhaps for devising much-needed new therapeutic solutions.
